# Editorial: Metagenomic insights into microbial communities in fruits and vegetable plants

**DOI:** 10.3389/fmicb.2026.1844864

**Published:** 2026-04-17

**Authors:** Chellappan Padmanabhan, Alina Puig

**Affiliations:** 1 United States Department of Agriculture, Animal and Plant Health Inspection Service, Plant Protection and Quarantine, Science and Technology, Plant Pathogen Confirmatory Diagnostics Laboratory, Laurel, MD, United States; 2 United States Department of Agriculture, Agricultural Research Service, Foreign Disease-Weed Science Research Unit, Fort Detrick, MD, United States

**Keywords:** diagnostics, fruits and vegetables, high-throughput sequencing, metagenome, microbiome, plant-pathogen interaction, rhizosphere, Viroscope

Metagenomics makes it possible to study all organisms present in a plant at a given time through direct sequencing, opening new avenues of investigation in the field of plant pathology. It originally emerged as a method to test for pathogens that could not be cultured and has since been incorporated into numerous research applications. These include the discovery of novel viruses, characterizing the microbial communities of crop plants, and understanding the interactions between plants and microbes (including pathogens), along with how microbial communities are shaped by external factors. Despite its potential, the use of metagenomics has been limited by the high cost of sequencing and the lack of necessary bioinformatics expertise (Roman-Reyna and Crandall, 2024), thus hindering important research on microbial communities in crop plants.

This research topic covers metagenomic insights into microbial communities in plants through the following contributions: “Viroscope-Advanced Cloud-Based Virus Diagnosis,” “Perspectives on High-Throughput Sequencing in Mexico,” “Wheat Microbial Communities Influenced by *Puccinia striiformis*,” “Nitrogen Fertilizer-Mediated Enrichment of Microbes to Improve Pepino (*Solanum muricatum*),” “Rhizosphere Microbiome and Metabolic Adaptation,” and “Detection of Active Bacterial Pathogens in Plants.”

## Viroscope™: advanced cloud-based virus diagnosis

Plant pathogens, particularly viruses and viroids, are highly destructive, accounting for approximately 50% of the diseases worldwide and over 30 billion USD in annual economic losses (Rodríguez-Verástegui et al., 2022). For example, the citrus tristeza virus (CTV) destroyed over 100 million citrus trees (Folimonova and Sun, 2022), and the tomato brown rugose fruit virus (ToBRFV) caused yield loss of up to 70% (Oladokun et al., 2019). Current import practices rely on strict phytosanitary customs, coupling conventional detection methods with visual inspection and traditional bioassays, such as enzyme-linked immunosorbent assays (ELISA). The concern with ELISA assays is that they are time-consuming and have high false negative rates and cross-reactivity with closely related species. Although PCR-based assays are the gold standard for the detection of known viruses, they require prior genomic information and therefore cannot be applied to novel viruses. To address these difficulties, high-throughput sequencing (HTS) technology offers the best methodology for the collective identification of known and novel viruses. Despite its advantages, HTS-based virus diagnosis is dependent on bioinformatic expertise and computational infrastructure, which has limited its widespread adoption. Morgante et al. developed Viroscope, which addresses the analytical challenges of HTS via an advanced Cloud service that leverages HTS for highly sensitive identification of low- abundance viruses and viroids.

## Perspectives on high-throughput sequencing in Mexico

High-throughput sequencing (HTS) has numerous applications, including pathogen detection and virome characterization. The article by Pacheco-Dorantes et al. summarized the current status of HTS in Mexico for identifying plant viruses and viroids and describes how it has revolutionized the field of plant virology in the country. Plant virology research in Mexico began in the mid-1900s and has expanded in scope and complexity as new technologies have become available. Until recently, diagnostic techniques were limited to the detection of known viruses; however, HTS does not require prior knowledge of the target. Since the introduction of this method less than a decade ago, 86 viruses/viroids have been detected in key crops such as corn, beans, grapes, and tomatoes in Mexico. This has led to an improved understanding of the viromes of these crops and has made it possible to describe and characterize previously unknown viruses. The authors theorized that HTS will enable researchers to develop a comprehensive understanding of viral populations, including complex co-infections that impact disease epidemiology.

## Wheat microbial communities influenced by *Puccinia striiformis*

When pathogenic microbes interact with plants, the associated microbial community may change dynamically. For instance, plant-associated microorganisms are actively involved in the plant's nutritional metabolism, and they can enable plants to take up essential nutrients, such as nitrogen, potassium, and phosphorus, from the soil. In response to infection by a pathogen, plants are directly enriched with more beneficial microorganisms, which are of crucial significance for improving plant disease resistance. However, some microorganisms are suppressed by highly dominant pathogens, which can grow faster and cause disease. This article detailed the research into understanding the composition and abundance of plant microbiomes when disrupted by disease-causing pathogens in the host. Several pathogen species cause diseases in crop plants, such as wheat. The wheat stripe rust disease is a significant threat to wheat production worldwide and is caused by the airborne fungus *Puccinia striiformis*. Gao et al. revealed the responses of wheat microbial communities in different niches—the phyllosphere and rhizosphere—to *P. striiformis* infection. The authors identified variations in certain microbial communities across a diverse set of wheat varieties. Highly disease-resistant varieties demonstrate greater optimization of microbial community functions that enhance disease resistance.

## Nitrogen fertilizer-mediated enrichment of microbes to improve pepino (*Solanum muricatum*)

In agricultural fields, the continuous application of chemical fertilizers promotes the accumulation of excess nutrients in the soil, disrupting the diversity, composition, and metabolic functions of soil microbial communities, which are negatively correlated with crop yield (Zhou et al., 2023). Pepino (*Solanum muricatum*) is a perennial herbaceous species in the Solanaceae family. Its fruit has nutritional value and contains significant levels of minerals, such as calcium, phosphorus, and potassium, along with ascorbic acid (vitamin C), and has potential health benefits, including anti-inflammatory, anti-cancer, and diabetes management properties. In recent years, pepino has emerged as a high-value crop. The influence of varying nitrogen fertilizers on pepino rhizosphere soil biochemical properties, plant growth, and fruit quality has been researched. Utilizing various nitrogen rates (e.g., N300, N225, N150, N75, and N0), it was determined that a nitrogen application rate of 150~225 kg-ha-1 is best for pepino production. Importantly, nitrogen fertilization indirectly increases pepino productivity by stimulating urease and nitrate reductase activities, and enhancing microbiota: Nitrosomonas, Opitutus, Ensifer, Mesorhizobium, and so on, to facilitate soil nutrient mobilization for plant growth and fruit nutrients (Su et al.).

## Rhizosphere microbiome and metabolic adaptation

Rhizosphere microbiomes include a diverse community of bacteria, fungi, and several other microbes. They are pivotal to plant health, nutrient cycling, and soil ecosystem stability. Microbes help plants access nutrients and fight pathogens while plants provide microbes with energy, significantly boosting plant health, growth, and disease resistance. Radish (*Raphanus sativus* L.) is an economically important vegetable crop that relies on its root-associated microbiome for stress resilience and nutrient acquisition (Kravchenko et al., 2022). Studies have shown that biodegradable mulches, such as polybutylene adipate terephthalate/polylactic acid (PBAT/PLA) and humic acid (HA)-modified PBAT/PLA films, are increasingly being used in sustainable agriculture and to enrich microbial activity. However, their effects on the structure and function of the plant rhizosphere microbial community have not been studied systematically. One of these research articles reported on the combined use of 16S rRNA/ITS sequencing and KEGG pathway analysis to characterize the Alpine radish rhizosphere microbiome and evaluate how the PBAT/PLA biodegradable multi-film modulates microbial community structure and shifts microbial metabolic activity. The results revealed that the incorporation of HA into PBAT/PLA biodegradable mulch films has dual benefits in both enriching functional microbial taxa and stimulating metabolic pathways essential for adaptation (Zhong et al.).

## Detection of active bacterial pathogens in plants

A study on *Xylella fastidiosa* subsp. *pauca* (Xfp), the causal agent of olive quick decline, used transcriptomic data from diseased trees to understand the interactions between the plant host and the bacterial pathogen during the infection process. This research identified a gene transcript, cvaC-1, which is generated during active bacterial multiplication and could serve as a valuable target for diagnostic tests. RT-qPCRs targeting cvaC-1 were even able to detect the pathogen early in the infection process, when levels of the pathogen are still very low and difficult to detect with existing diagnostic tests. In addition to this, RT-qPCRs targeting cvaC-1 have the benefit of differentiating between actively growing and dormant, or non-viable, bacterial fragments. This finding addresses the diagnostic challenge caused by Xfp due to non-viable fragments of the bacterium remaining detectable in plants (Serena Amoia et al.).
